# Supplementary material for: Barriers and Facilitators to Smoking Cessation Among University Students: A Scoping Review
Source: Int J Environ Res Public Health. 2025 Jun 17;22(6):947. doi: 10.3390/ijerph22060947 (PMC12193249; doi:10.3390/ijerph22060947)
Supplement: Supplementary file 1 [file ijerph-22-00947-s001.zip › Table S2. Modified JBI data extraction tool.pdf]

**Table S2.** Modified JBI data extraction tool

| | |
|---|---|
| **Author/s** | |
| **Year of Publication** | |
| **Country** | |
| **Aim** | |
| **Research Design** | |
| **Data Collection Method** | |
| **Sample Size** | |
| **Population** | |
| **Setting** | |
| **Smoking Behaviour** | |
| **Key Findings** | |
