# Supplementary material for: Barriers and Facilitators to Smoking Cessation Among University Students: A Scoping Review
Source: Int J Environ Res Public Health. 2025 Jun 17;22(6):947. doi: 10.3390/ijerph22060947 (PMC12193249; doi:10.3390/ijerph22060947)
Supplement: Supplementary file 1 [file ijerph-22-00947-s001.zip › Text S1 Search strategy.pdf]

Text S1. Search strategy for all databases.

Database:
Ovid MEDLINE(R) ALL <1946 to February 17, 2025>

#	Query	Results from 18 Feb 2025
1	exp Smoking Cessation/	34,215
2	"Smoking Cessation".mp. [mp=title, book title, abstract, original title, name of substance word, subject heading word, floating sub-heading word, keyword heading word, organism supplementary concept word, protocol supplementary concept word, rare disease supplementary concept word, unique identifier, synonyms, population supplementary concept word, anatomy supplementary concept word]	47,991
3	"Tobacco Cessation".mp. [mp=title, book title, abstract, original title, name of substance word, subject heading word, floating sub-heading word, keyword heading word, organism supplementary concept word, protocol supplementary concept word, rare disease supplementary concept word, unique identifier, synonyms, population supplementary concept word, anatomy supplementary concept word]	3,482
4	"Nicotine Cessation".mp. [mp=title, book title, abstract, original title, name of substance word, subject heading word, floating sub-heading word, keyword heading word, organism supplementary concept word, protocol supplementary concept word, rare disease supplementary concept word, unique identifier, synonyms, population supplementary concept word, anatomy supplementary concept word]	129
5	"Quit Smoking".mp. [mp=title, book title, abstract, original title, name of	8,099

	substance word, subject heading word, floating sub-heading word, keyword heading word, organism supplementary concept word, protocol supplementary concept word, rare disease supplementary concept word, unique identifier, synonyms, population supplementary concept word, anatomy supplementary concept word]	
6	Enabler*.mp. [mp=title, book title, abstract, original title, name of substance word, subject heading word, floating sub-heading word, keyword heading word, organism supplementary concept word, protocol supplementary concept word, rare disease supplementary concept word, unique identifier, synonyms, population supplementary concept word, anatomy supplementary concept word]	7,628
7	Barrier*.mp. [mp=title, book title, abstract, original title, name of substance word, subject heading word, floating sub-heading word, keyword heading word, organism supplementary concept word, protocol supplementary concept word, rare disease supplementary concept word, unique identifier, synonyms, population supplementary concept word, anatomy supplementary concept word]	486,526
8	Facilitator*.mp. [mp=title, book title, abstract, original title, name of substance word, subject heading word, floating sub-heading word, keyword heading word, organism supplementary concept word, protocol supplementary concept word, rare disease supplementary concept word, unique identifier, synonyms, population supplementary concept word, anatomy supplementary concept word]	47,183
9	Obstacle*.mp. [mp=title, book title, abstract, original title, name of	75,540

	substance word, subject heading word, floating sub-heading word, keyword heading word, organism supplementary concept word, protocol supplementary concept word, rare disease supplementary concept word, unique identifier, synonyms, population supplementary concept word, anatomy supplementary concept word]	
10	Challenge*.mp. [mp=title, book title, abstract, original title, name of substance word, subject heading word, floating sub-heading word, keyword heading word, organism supplementary concept word, protocol supplementary concept word, rare disease supplementary concept word, unique identifier, synonyms, population supplementary concept word, anatomy supplementary concept word]	1,113,914
11	Motivat*.mp. [mp=title, book title, abstract, original title, name of substance word, subject heading word, floating sub-heading word, keyword heading word, organism supplementary concept word, protocol supplementary concept word, rare disease supplementary concept word, unique identifier, synonyms, population supplementary concept word, anatomy supplementary concept word]	243,367
12	Engag*.mp. [mp=title, book title, abstract, original title, name of substance word, subject heading word, floating sub-heading word, keyword heading word, organism supplementary concept word, protocol supplementary concept word, rare disease supplementary concept word, unique identifier, synonyms, population supplementary concept word, anatomy supplementary concept word]	304,978
13	Adher*.mp. [mp=title, book title, abstract, original title, name of	316,499

	substance word, subject heading word, floating sub-heading word, keyword heading word, organism supplementary concept word, protocol supplementary concept word, rare disease supplementary concept word, unique identifier, synonyms, population supplementary concept word, anatomy supplementary concept word]	
14	Undergraduate*.mp. [mp=title, book title, abstract, original title, name of substance word, subject heading word, floating sub-heading word, keyword heading word, organism supplementary concept word, protocol supplementary concept word, rare disease supplementary concept word, unique identifier, synonyms, population supplementary concept word, anatomy supplementary concept word]	81,885
15	"College Student*".mp. [mp=title, book title, abstract, original title, name of substance word, subject heading word, floating sub-heading word, keyword heading word, organism supplementary concept word, protocol supplementary concept word, rare disease supplementary concept word, unique identifier, synonyms, population supplementary concept word, anatomy supplementary concept word]	31,899
16	"University Student*".mp. [mp=title, book title, abstract, original title, name of substance word, subject heading word, floating sub-heading word, keyword heading word, organism supplementary concept word, protocol supplementary concept word, rare disease supplementary concept word, unique identifier, synonyms, population supplementary concept word, anatomy supplementary concept word]	24,825
17	("Higher Education Institution" and Student*).mp. [mp=title, book title,	344

	abstract, original title, name of substance word, subject heading word, floating sub-heading word, keyword heading word, organism supplementary concept word, protocol supplementary concept word, rare disease supplementary concept word, unique identifier, synonyms, population supplementary concept word, anatomy supplementary concept word]	
18	(HEI and Student*).mp. [mp=title, book title, abstract, original title, name of substance word, subject heading word, floating sub-heading word, keyword heading word, organism supplementary concept word, protocol supplementary concept word, rare disease supplementary concept word, unique identifier, synonyms, population supplementary concept word, anatomy supplementary concept word]	232
19	Postgraduate*.mp. [mp=title, book title, abstract, original title, name of substance word, subject heading word, floating sub-heading word, keyword heading word, organism supplementary concept word, protocol supplementary concept word, rare disease supplementary concept word, unique identifier, synonyms, population supplementary concept word, anatomy supplementary concept word]	24,035
20	1 or 2 or 3 or 4 or 5	51,240
21	6 or 7 or 8 or 9 or 10 or 11 or 12 or 13	2,330,988
22	14 or 15 or 16 or 17 or 18	130,956
23	20 and 21 and 22	193

Database:
Embase <1974 to 2025 Week 07>

#	Query	Results from 18 Feb 2025
1	exp Smoking Cessation/	73,677
2	"Smoking Cessation".mp. [mp=title, abstract, heading word, drug trade name, original title, device manufacturer, drug manufacturer, device trade name, keyword heading word, floating subheading word, candidate term word]	82,314
3	"Tobacco Cessation".mp. [mp=title, abstract, heading word, drug trade name, original title, device manufacturer, drug manufacturer, device trade name, keyword heading word, floating subheading word, candidate term word]	4,601
4	"Nicotine Cessation".mp. [mp=title, abstract, heading word, drug trade name, original title, device manufacturer, drug manufacturer, device trade name, keyword heading word, floating subheading word, candidate term word]	181
5	"Quit Smoking".mp. [mp=title, abstract, heading word, drug trade name, original title, device manufacturer, drug manufacturer, device trade name, keyword heading word, floating subheading word, candidate term word]	10,651
6	Enabler*.mp. [mp=title, abstract, heading word, drug trade name, original title, device manufacturer, drug manufacturer, device trade name, keyword heading word, floating subheading word, candidate term word]	8,981

7	Barrier*.mp. [mp=title, abstract, heading word, drug trade name, original title, device manufacturer, drug manufacturer, device trade name, keyword heading word, floating subheading word, candidate term word]	626,420
8	Facilitator*.mp. [mp=title, abstract, heading word, drug trade name, original title, device manufacturer, drug manufacturer, device trade name, keyword heading word, floating subheading word, candidate term word]	57,908
9	Obstacle*.mp. [mp=title, abstract, heading word, drug trade name, original title, device manufacturer, drug manufacturer, device trade name, keyword heading word, floating subheading word, candidate term word]	90,261
10	Challenge*.mp. [mp=title, abstract, heading word, drug trade name, original title, device manufacturer, drug manufacturer, device trade name, keyword heading word, floating subheading word, candidate term word]	1,338,039
11	Motivat*.mp. [mp=title, abstract, heading word, drug trade name, original title, device manufacturer, drug manufacturer, device trade name, keyword heading word, floating subheading word, candidate term word]	289,641
12	Engag*.mp. [mp=title, abstract, heading word, drug trade name, original title, device manufacturer, drug manufacturer, device trade name, keyword heading word, floating subheading word, candidate term word]	388,591
13	Adher*.mp. [mp=title, abstract, heading word, drug trade name, original title,	426,199

	device manufacturer, drug manufacturer, device trade name, keyword heading word, floating subheading word, candidate term word]	
14	Undergraduate*.mp. [mp=title, abstract, heading word, drug trade name, original title, device manufacturer, drug manufacturer, device trade name, keyword heading word, floating subheading word, candidate term word]	74,518
15	"College Student*".mp. [mp=title, abstract, heading word, drug trade name, original title, device manufacturer, drug manufacturer, device trade name, keyword heading word, floating subheading word, candidate term word]	42,711
16	"University Student*".mp. [mp=title, abstract, heading word, drug trade name, original title, device manufacturer, drug manufacturer, device trade name, keyword heading word, floating subheading word, candidate term word]	31,579
17	("Higher Education Institution" and Student*).mp. [mp=title, abstract, heading word, drug trade name, original title, device manufacturer, drug manufacturer, device trade name, keyword heading word, floating subheading word, candidate term word]	397
18	(HEI and Student*).mp. [mp=title, abstract, heading word, drug trade name, original title, device manufacturer, drug manufacturer, device trade name, keyword heading word, floating subheading word, candidate term word]	318
19	Postgraduate*.mp. [mp=title, abstract, heading word, drug trade name, original title, device manufacturer, drug	47,265

	manufacturer, device trade name, keyword heading word, floating subheading word, candidate term word]	
20	1 or 2 or 3 or 4 or 5	84,279
21	6 or 7 or 8 or 9 or 10 or 11 or 12 or 13	2,886,890
22	14 or 15 or 16 or 17 or 18	137,460
23	20 and 21 and 22	237

Database:
APA PsycInfo <1806 to February 2025 Week 2>

#	Query	Results from 18 Feb 2025
1	exp Smoking Cessation/	15,723
2	"Smoking Cessation".mp. [mp=title, abstract, heading word, table of contents, key concepts, original title, tests & measures, mesh word]	21,212
3	"Tobacco Cessation".mp. [mp=title, abstract, heading word, table of contents, key concepts, original title, tests & measures, mesh word]	1,407
4	"Nicotine Cessation".mp. [mp=title, abstract, heading word, table of contents, key concepts, original title, tests & measures, mesh word]	67
5	"Quit Smoking".mp. [mp=title, abstract, heading word, table of contents, key concepts, original title, tests & measures, mesh word]	3,993
6	Enabler*.mp. [mp=title, abstract, heading word, table of contents, key concepts, original title, tests & measures, mesh word]	3,132
7	Barrier*.mp. [mp=title, abstract, heading word, table of contents, key concepts, original title, tests & measures, mesh word]	111,705
8	Facilitator*.mp. [mp=title, abstract, heading word, table of contents, key concepts, original title, tests & measures, mesh word]	23,076

9	Obstacle*.mp. [mp=title, abstract, heading word, table of contents, key concepts, original title, tests & measures, mesh word]	25,165
10	Challenge*.mp. [mp=title, abstract, heading word, table of contents, key concepts, original title, tests & measures, mesh word]	290,548
11	Motivat*.mp. [mp=title, abstract, heading word, table of contents, key concepts, original title, tests & measures, mesh word]	252,952
12	Engag*.mp. [mp=title, abstract, heading word, table of contents, key concepts, original title, tests & measures, mesh word]	291,202
13	Adher*.mp. [mp=title, abstract, heading word, table of contents, key concepts, original title, tests & measures, mesh word]	55,067
14	Undergraduate*.mp. [mp=title, abstract, heading word, table of contents, key concepts, original title, tests & measures, mesh word]	130,655
15	"College Student*".mp. [mp=title, abstract, heading word, table of contents, key concepts, original title, tests & measures, mesh word]	221,531
16	"University Student*".mp. [mp=title, abstract, heading word, table of contents, key concepts, original title, tests & measures, mesh word]	43,903
17	("Higher Education Institution" and Student*).mp. [mp=title, abstract, heading word, table of contents, key concepts, original title, tests & measures, mesh word]	577
18	(HEI and Student*).mp. [mp=title, abstract, heading word, table of contents, key concepts, original title, tests & measures, mesh word]	172
19	Postgraduate*.mp. [mp=title, abstract, heading word, table of contents, key concepts, original title, tests & measures, mesh word]	9,305
20	1 or 2 or 3 or 4 or 5	22,058
21	6 or 7 or 8 or 9 or 10 or 11 or 12 or 13	900,572
22	14 or 15 or 16 or 17 or 18	298,424
23	20 and 21 and 22	162

Database:

Scopus

#	Query	Results
1	TITLE-ABS-KEY (undergraduate* OR "College Student*" OR "University Student*" OR ("Higher Education Institution*" AND student*) OR (hei AND student*) OR postgraduate*)	486,102
2	TITLE-ABS-KEY (enabler* OR barrier* OR facilitator* OR obstacle* OR challenge* OR motivat* OR engag* OR adher*)	6,607,559
3	TITLE-ABS-KEY ("Smoking Cessation" OR "Tobacco Cessation" OR "Nicotine Cessation" OR "Quit Smoking")	79,747
4	1 AND 2 AND 3	326

Database:

Web of Science

#	Query	Results
1	TS=("Smoking Cessation" OR "Tobacco Cessation" OR "Nicotine Cessation" OR "Quit Smoking")	46,498
2	TS=(Enabler* OR Barrier* OR Facilitator* OR Obstacle* OR Challenge* OR Motivat* OR Engag* OR Adher*)	5,132,991
3	TS=(Undergraduate* OR "College Student*" OR "University Student*" OR ("Higher Education Institution*" AND Student*) OR (HEI AND Student*) OR Postgraduate*)	342,208
4	1 AND 2 AND 3	249

Database:

CINAHL via (EBSCO)

#	Query	Limiters/Expanders	Last Run Via	Results
S23	S20 AND S21 AND S22	Expanders - Apply equivalent subjectsSearch modes - Proximity	Interface - EBSCOhost Research DatabasesSearch Screen - Advanced SearchDatabase - CINAHL Ultimate	146
S22	S1 OR S2 OR S3 OR S4 OR S5	Expanders - Apply equivalent subjectsSearch modes - Proximity	Interface - EBSCOhost Research DatabasesSearch Screen - Advanced SearchDatabase - CINAHL Ultimate	31,817
S21	S6 OR S7 OR S8 OR S9 OR S10 OR S11 OR S12 OR S13	Expanders - Apply equivalent subjectsSearch modes - Proximity	Interface - EBSCOhost Research DatabasesSearch Screen - Advanced SearchDatabase - CINAHL Ultimate	644,798
S20	S14 OR S15 OR S16 OR S17 OR S18 OR S19	Expanders - Apply equivalent subjectsSearch modes - Proximity	Interface - EBSCOhost Research DatabasesSearch Screen - Advanced SearchDatabase - CINAHL Ultimate	71,201
S19	Postgraduate*	Expanders - Apply equivalent subjectsSearch modes - Proximity	Interface - EBSCOhost Research DatabasesSearch Screen - Advanced SearchDatabase - CINAHL Ultimate	7,672
S18	"Higher Education Institution" AND Student*	Expanders - Apply equivalent subjectsSearch modes - Proximity	Interface - EBSCOhost Research DatabasesSearch Screen - Advanced SearchDatabase - CINAHL Ultimate	382
S17	HEI AND Student*	Expanders - Apply equivalent subjectsSearch modes - Proximity	Interface - EBSCOhost Research DatabasesSearch Screen - Advanced SearchDatabase - CINAHL Ultimate	276
S16	"University Student*"	Expanders - Apply equivalent subjectsSearch modes - Proximity	Interface - EBSCOhost Research DatabasesSearch Screen - Advanced SearchDatabase - CINAHL Ultimate	28,717
S15	"College Student*"	Expanders - Apply equivalent subjectsSearch modes - Proximity	Interface - EBSCOhost Research DatabasesSearch Screen - Advanced	31,827

#	Query	Limiters/Expanders	Last Run Via	Results
			SearchDatabase - CINAHL Ultimate	
S14	Undergraduate*	Expanders - Apply equivalent subjectsSearch modes - Proximity	Interface - EBSCOhost Research DatabasesSearch Screen - Advanced SearchDatabase - CINAHL Ultimate	35,019
S13	Adher*	Expanders - Apply equivalent subjectsSearch modes - Proximity	Interface - EBSCOhost Research DatabasesSearch Screen - Advanced SearchDatabase - CINAHL Ultimate	93,710
S12	Engag*	Expanders - Apply equivalent subjectsSearch modes - Proximity	Interface - EBSCOhost Research DatabasesSearch Screen - Advanced SearchDatabase - CINAHL Ultimate	135,884
S11	Motivat*	Expanders - Apply equivalent subjectsSearch modes - Proximity	Interface - EBSCOhost Research DatabasesSearch Screen - Advanced SearchDatabase - CINAHL Ultimate	95,695
S10	Challenge*	Expanders - Apply equivalent subjectsSearch modes - Proximity	Interface - EBSCOhost Research DatabasesSearch Screen - Advanced SearchDatabase - CINAHL Ultimate	257,456
S9	Obstacle*	Expanders - Apply equivalent subjectsSearch modes - Proximity	Interface - EBSCOhost Research DatabasesSearch Screen - Advanced SearchDatabase - CINAHL Ultimate	16,791
S8	Facilitator*	Expanders - Apply equivalent subjectsSearch modes - Proximity	Interface - EBSCOhost Research DatabasesSearch Screen - Advanced SearchDatabase - CINAHL Ultimate	23,921
S7	Barrier*	Expanders - Apply equivalent subjectsSearch modes - Proximity	Interface - EBSCOhost Research DatabasesSearch Screen - Advanced SearchDatabase - CINAHL Ultimate	134,487
S6	Enabler*	Expanders - Apply equivalent subjectsSearch modes - Proximity	Interface - EBSCOhost Research DatabasesSearch Screen - Advanced SearchDatabase - CINAHL Ultimate	4,116

#	Query	Limiters/Expanders	Last Run Via	Results
S5	"Quit Smoking"	Expanders - Apply equivalent subjectsSearch modes - Proximity	Interface - EBSCOhost Research DatabasesSearch Screen - Advanced SearchDatabase - CINAHL Ultimate	17,339
S4	"Nicotine Cessation"	Expanders - Apply equivalent subjectsSearch modes - Proximity	Interface - EBSCOhost Research DatabasesSearch Screen - Advanced SearchDatabase - CINAHL Ultimate	43
S3	"Tobacco Cessation"	Expanders - Apply equivalent subjectsSearch modes - Proximity	Interface - EBSCOhost Research DatabasesSearch Screen - Advanced SearchDatabase - CINAHL Ultimate	1,973
S2	"Smoking Cessation"	Expanders - Apply equivalent subjectsSearch modes - Proximity	Interface - EBSCOhost Research DatabasesSearch Screen - Advanced SearchDatabase - CINAHL Ultimate	30,670
S1	(MH "Smoking Cessation")	Expanders - Apply equivalent subjectsSearch modes - Proximity	Interface - EBSCOhost Research DatabasesSearch Screen - Advanced SearchDatabase - CINAHL Ultimate	23,551
