# Supplementary material for: Barriers and Facilitators to Smoking Cessation Among University Students: A Scoping Review
Source: Int J Environ Res Public Health. 2025 Jun 17;22(6):947. doi: 10.3390/ijerph22060947 (PMC12193249; doi:10.3390/ijerph22060947)
Supplement: Supplementary file 1 [file ijerph-22-00947-s001.zip › Text S2. Excluded Studies.pdf]

Text S2. Excluded Studies

1. Studies that do not explicitly address barriers or facilitators to smoking cessation (n = 29)

1.1. Studies focused on smoking behaviour, prevalence, or perceptions (n = 16)

1. Al-Natour, Ahlam, Gordon Lee Gillespie, and Fatmeh Alzoubi. "“We cannot stop smoking”: Female university students' experiences and perceptions." *Applied Nursing Research* 61 (2021): 151477.
2. Sugiyatmi, Tri Astuti, Lukman Handoko, Alfrid Sentosa, Fitriyanti Fitriyanti, and Sri Mulyani. "Examining the onset and cessation patterns of active smoking among college students: an interpretative analysis." *Healthcare in Low-resource Settings* 12, no. s1 (2024).
3. Prokhorov, Alexander V., Carla Warneke, Carl de Moor, Karen M. Emmons, Mary Mullin Jones, Carol Rosenblum, Karen Suchanek Hudmon, and Ellen R. Gritz. "Self-reported health status, health vulnerability, and smoking behavior in college students: implications for intervention." *Nicotine & Tobacco Research* 5, no. 4 (2003): 545-552.
4. Kim, Hee Jeong. "Smoking Behavior, Smoking Motivation, Stress, Physical Condition and Health Promotion Behavior of College Students with No smoking Intention." *Medico-legal Update* 19, no. 2 (2019).
5. Tadjoedin, Irfan Hasyim, Adityo Wibowo, and Triya Damayanti. "Determining Factors for Smoking Habits and FeNO Levels in Male College Student Smokers." (2024).
6. Maksimovic, Jadranka M., Aleksandar D. Pavlovic, Hristina D. Vlajinac, Isidora S. Vujcic, Sandra B. Sipetic Grujicic, Slavica R. Maris, Milos Z. Maksimovic, Milan R. Obrenovic, and Ivana I. Kavecan. "Factors associated with smoking habits among undergraduate medical students: a cross-sectional study." *Journal of Substance Use* 28, no. 2 (2023): 206-210.
7. Aoike, Makoto, Yukihiro Mori, Kiyoshi Hotta, Yukihiro Shigeno, Yuka Aoyama, Mamoru Tanaka, Hana Kouzai, Hatsumi Kawamura, Masato Tsurudome, and Morihiro Ito. "Evaluation of Japanese university students' perception of smoking, interest in quitting, and smoking behavior: An examination and public health challenges during the COVID-19 pandemic." *Drug Discoveries & Therapeutics* 16, no. 3 (2022): 118-123.
8. Raddaha, Ahmad Hasan Abu, and Amirat Ali Al-Sabeely. "Female nursing students’ knowledge, attitudes, beliefs and behaviors toward smoking: A cross-sectional study in Saudi Arabia." *Nursing Practice Today* 9, no. 4 (2022): 303-313.
9. Berg, Carla J., Pamela M. Ling, Rashelle B. Hayes, Erin Berg, Nikki Nollen, Eric Nehl, Won S. Choi, and Jasjit S. Ahluwalia. "Smoking frequency among current college student smokers: distinguishing characteristics and factors related to readiness to quit smoking." *Health education research* 27, no. 1 (2012): 141-150.
10. Filippi, Melissa K., Charlotte McCloskey, Chandler Williams, Julia White Bull, Won S. Choi, K. Allen Greiner, and Christine M. Daley. "Perceptions, barriers, and suggestions for creation of a tobacco and health website among American Indian/Alaska Native college students." *Journal of Community Health* 38 (2013): 486-491.
11. Elkalmi, Ramadan M., Ramez M. Alkoudmani, Tarek M. Elsayed, Akram Ahmad, and Muhammad Umair Khan. "Effect of religious beliefs on the smoking behaviour of university

students: quantitative findings from Malaysia." *Journal of religion and health* 55 (2016): 1869-1875.
12. Saravanan, Coumaravelou, and Imran Heidhy. "Psychological problems and psychosocial predictors of cigarette smoking behavior among undergraduate students in Malaysia." *Asian Pacific Journal of Cancer Prevention* 15, no. 18 (2014): 7629-7634.
13. Lee, Hyoung S., Delwyn Catley, and Kari Jo Harris. "Improving understanding of the quitting process: psychological predictors of quit attempts versus smoking cessation maintenance among college students." *Substance use & misuse* 49, no. 10 (2014): 1332-1339.
14. Olson, Phayom Sookaneknun, Saithip Suttiruksa, Issara Chummalee, Theerapong Seesin, Rodchares Nithipaichit, Terdsak Promarak, Teabpaluck Sirithanawuthichai et al. "Evaluating smokers' opinions on smoking and customized cessation in a Thailand University context: A qualitative study." *Tobacco Induced Diseases* 22 (2024).
15. Clark, Eileen, and Terence V. McCann. "The influence of friends on smoking commencement and cessation in undergraduate nursing students: A survey." *Contemporary Nurse* 27, no. 2 (2008): 185-193.
16. Oh, HyunSoo, BoAe Im, and WhaSook Seo. "Comparisons of the stages and psychosocial factors of smoking cessation and coping strategies for smoking cessation in college student smokers: Conventional cigarette smokers compared to dual smokers of conventional and e-cigarettes." *Japan Journal of Nursing Science* 16, no. 4 (2019): 345-354.

1.2. Intervention studies without analysis of barriers/facilitators (n = 7)

1. Hofmeyr, Andre, Harold Kincaid, and Olivia Rusch. "Incentivizing university students to quit smoking: a randomized controlled trial of a contingency management intervention in a developing country." *The American Journal of Drug and Alcohol Abuse* 46, no. 1 (2020): 109-119.
2. Jorayeva, Anna, S. Lee Ridner, Lynne Hall, Ruth Staten, and Kandi L. Walker. "A novel text message-based motivational interviewing intervention for college students who smoke cigarettes." *Tobacco Prevention & Cessation* 3 (2017).
3. Pardavila-Belio, Miren I., Cristina García-Vivar, Adriano Marçal Pimenta, Ana Canga-Armayor, Sara Pueyo-Garrigues, and Navidad Canga-Armayor. "Intervention study for smoking cessation in Spanish college students: pragmatic randomized controlled trial." *Addiction* 110, no. 10 (2015).
4. Salmani, Babac, and Harry Prapavessis. "Using a protection motivation theory framework to reduce vaping intention and behaviour in Canadian university students who regularely vape: a randomized controlled trial." *Journal of Health Psychology* 28, no. 9 (2023): 832-845.
5. Eavers, Erika R., Melissa A. Berry, and Dario N. Rodriguez. "The effects of counterfactual thinking on college students' intentions to quit smoking cigarettes." *Current Research in Social Psychology* 24 (2015): 66-77.
6. Orsal, Ozgul, and Ayse Ergun. "The effect of peer education on decision-making, smoking-promoting factors, self-efficacy, addiction, and behavior change in the process of quitting smoking of young people." *Risk Management and Healthcare Policy* (2021): 925-945.
7. Abdelaal, Ashraf Abdelaal Mohamed, and Gihan Mousa. "Long-term effect of telling the lung age on smoking quit rate in undergraduate smokers: a one-year follow-up randomized controlled study." *Physiotherapy Quarterly* 30, no. 3 (2022): 44-50.

1.3. Studies focused on theory or motivation, without barriers/facilitators analysis (n = 5)

1. Berg, Carla J. "Nondaily Smoking Cessation motivation for young adults: Scale development and validation." *Journal of Smoking Cessation* 8, no. 2 (2013): 97-105.
2. Gemmell, Leigh, and Carlo C. DiClemente. "Styles of physician advice about smoking cessation in college students." *Journal of American College Health* 58, no. 2 (2009): 113-119.
3. Lee, Hyoung S., Delwyn Catley, and Kari Jo Harris. "A comparison of autonomous regulation and negative self-evaluative emotions as predictors of smoking behavior change among college students." *Journal of health psychology* 17, no. 4 (2012): 600-609.
4. Bowen, Sarah, and Andrew S. Kurz. "Smoking, nicotine dependence, and motives to quit in Asian American versus Caucasian college students." *Nicotine & Tobacco Research* 14, no. 10 (2012): 1235-1240.
5. Sharma, Manoj, Kavita Batra, Ravi Batra, Chia-Liang Dai, Traci Hayes, Melinda J. Ickes, and Tejinder Pal Singh. "Assessing the testability of the multi-theory model (MTM) in predicting vaping quitting behavior among young adults in the United States: a cross-sectional survey." *International Journal of Environmental Research and Public Health* 19, no. 19 (2022): 12139.

1.4. Duplicated study (n = 1)

1- Kim, Hee Jeong. "Smoking Behavior, Smoking Motivation, Stress, Physical Condition and Health Promotion Behavior of College Students with No smoking Intention." *Medico-legal Update* 19, no. 2 (2019).

2. Studies do not focus on university students or include other populations alongside university students (n = 2)

1- Dobbs, P. D., Y. Lu, C. M. Dunlap, K. V. Newcombe, C. M. Baer, E. Hodges, and M. K. Cheney. "Young adults' intention to quit using JUUL." *Drug and Alcohol Dependence* 218 (2021): 108399.

2- Poreddi, Vijayalakshmi, Sailaxmi Gandhi, Rama Chandra, Andrew Wilson, and Suresh Bada Math. "Smoking cessation support: Indian nursing students' practices, attitudes and perceived barriers." *British Journal of Nursing* 24, no. 22 (2015): 1120-1128.

3. The full text is in a language other than English or Arabic (n = 1)

1- Shamsipour, Mansour, R. Korani Bahador, Asghar Mohammadpoorasl, and Asiah Mansouri. "Smoking prevalence and associated factors to quit among Tabriz dormitory university medical students, Tabriz, Iran." (2012): 20123144989.
